# Annotations

**Published:** 1891-06-06

**Authors:** 


### Annotations.

**The Laureate and the Nurses.** It is a unique occasion on which the most illustrious of living poets and men of letters has promised to break the self-imposed silence of venerable age. Lord Tennyson desires to show his gratitude, and to mark his appreciation of the rare value of trained and skilful women in the sick room. The Hospital is a paper which devotes itself partly to the interests of nurses, but, as its name implies, it embraces a wider range of activity than the nursing world. It can, therefore, quite independently, and to a certain extent as an outsider, congratulate nurses on the distinguished advocate they have gained for their cause. There is not a reader in the world but will be pleased to hear even a single note of song from the Laureate's muse. This country is not one to forget its famous men as they advance in years. On the contrary, the longer they live the deeper becomes our reverence for them, and the warmer our love. Even political opponents think in gentler strain and speak in milder tones of enemies whom age has bent, and whose hair is whitened by the frosts of many winters. Nursing has a peculiar part to play in the care of aged men. The man of eighty is often more beautiful than the child of eight, and sometimes needs still more tenderness and care. The flame of life in the infant is nursed and tended until it grows strong enough to burn steadily by its own inherent energy; the flame of life in the aged is likewise nursed and tended, lest some unlucky accident should suddenly extinguish it before its time. Here it is that the constant and watchful nurse finds her opportunity. She learns by daily experience to know not only all the highways, but the byeways and secret hiding places of her patient's constitution. To a trained nurse Lord Tennyson owes much of the health and comfort of his present happy condition. The world hopes that he may live long to be one of its brightest ornaments, as well as one of the strongest pillars of its virtue.

**Vegetarianism and Influenza.** The Secretary of the Vegetarian Society writes to us to claim for fruit and vegetables great potency in the prevention of influenza. Not only does uncooked fruit possess much virtue as a preventive, says this amiable enthusiast, but vegetables well-chosen are declared to be most useful in the treatment of the disease. The present writer has had personal experience both of influenza and of vegetarianism. He had a severe attack of the former about fifteen months ago, and a slight attack of the latter about nine months since. He speaks from experience and without prejudice. Personally, he found influenza to be a serious, distressing, and enfeebling malady. Vegetarianism he found to be rather less serious, though both distressing and enfeebling: distressing in that a whole meal of vegetables could with great difficulty be digested; and enfeebling in that it was quite impossible either to eat or to assimilate sufficient to maintain even moderate strength. He gave up the influenza gradually and with pleasure, but he quarrelled with vegetarianism suddenly and with satisfaction mixed with disgust. It seems a pity to write strongly in opposition to the views of a well-meaning lady; but, unfortunately, neither the lady nor anybody else seems inclined to believe at all in convictions that are not energetically expressed. We have great faith in vegetables, but not in vegetarianism; and much faith in fruit, but none in exclusively frugivorous men. The plain truth is that in a climate like our own, and still more in colder countries, pure vegetarianism is pure folly, except for a very few abnormal individuals. In Africa, of which we know something, vegetarianism prevails to a considerable extent. The natives eat flesh much more rarely than we do; though they, like wise people, eat it whenever they can get it. But what pot-bellied and ungainly creatures many of them are compared with Englishmen of the same age. Similarly, the "mild Hindoo" stuffs himself with rice; and how feeble in body and effeminate in mind he appears when compared with his British conqueror. Your frog-eating Frenchman, too, who takes a month to eat as much beef as an Englishman would eat in a day—what can he do when put on the track with English cyclists? The other day, at the great international cycling race in Paris, five English were entered against more than thirty of French and other nationalities. Of the five, four of our fellow-countrymen passed the winning post some hours before the first Frenchman put in an appearance. As for cricket and football, the frog-eater shivers at the mere mention of them. He would fly from a swift round-bowler like a fright-mad hare before a thoroughbred greyhound; while a scrimmage of the Rugby game would kill him outright. No, no! Miss Yates, your vegetarians, like babies and third wives, are very well in their place. But the strong man who has to front the world boldly in a stern climate, must have mutton and beef, with an occasional variation into turtle soup, and even green fat. Your true vegetarian is a very milk-and-water creature; and milk and water is not satisfactory to anybody except to milkmen of the slyer sort. The "roast beef of Old England" will stand many a siege from the washed-out members of the Vegetarian Society.

**Personal Troubles.** "The refuge you are needing from the personal troubles," says George Eliot, in "*Daniel Deronda,*" "is the higher, the religious life, which holds an enthusiasm for something more than our own appetites and vanities. The few may find themselves in it simply by an elevation of feeling, but for us who have to struggle for our wisdom, the higher life must be a region in which the affections are clad with knowledge." "Personal trouble" is not always of mental or emotional origin. Very often the cause of it is physiological and physical. Somebody has said that those young men fall in love most enthusiastically and passionately whose livers are most liable to get out of order. Cromwell, long before he became a soldier and an antagonist of the divine right of kings, was much given to profound spiritual depressions and wrestlings with what he held to be unworthiness and sin. Similarly, Cowper, the greatest poet of evangelicalism, except Charles Wesley, endured many mental martyrdoms which probably had physical origins. It is very likely that if Cromwell and Cowper had lived in our times they would have been persuaded to seek medical advice, and it is not improbable that if they had now and again been ordered a course of blue pill and Æsculap water, together with an occasional change to a mountainous region and a bracing air, they might have been much less troubled with spiritual difficulties. This is one aspect of the question of personal trouble. But there is another aspect equally true to fact and equally important. Many personal troubles which appear to be of a physical kind have mental and emotional sources. The doctor who could really lay his finger upon mental and emotional troubles with precision would very often succeed in effecting cures where he now fails. George Eliot, in the passage we have quoted at the beginning, proves herself a true physician. The cure of a certain class of personal troubles is certainly "the higher, the religious life which holds an enthusiasm for something more than our own appetites and vanities." The physiologist and the physician are quite familiar with persons who consistently "eat out their own hearts" by unceasingly dwelling upon their own personal experiences. It is just as true that no man can be well who has not something to care for and love outside himself as that he cannot be happy. The mental and emotional activities must be exercised on objects external to self. That which theologians call the love of God and the love of man is one of the primest of all the essentials of physical as well as of mental health. We make no apology whatever for dealing frequently with subjects of this kind. The physician who takes no account of the mental and emotional condition of his patients does not understand more than half his business.

